# Supplementary material for: Association between oral microbiome and seven types of cancers in East Asian population: a two-sample Mendelian randomization analysis
Source: Front Mol Biosci. 2023 Nov 21;10:1327893. doi: 10.3389/fmolb.2023.1327893 (PMC10702768; doi:10.3389/fmolb.2023.1327893)
Supplement: Supplementary file 1 [file DataSheet1.ZIP › Supplementary Materials/MR plots for tongue/tongue═╝/Lung cancer/pheno.1320_to_lung cancer_forest.pdf]

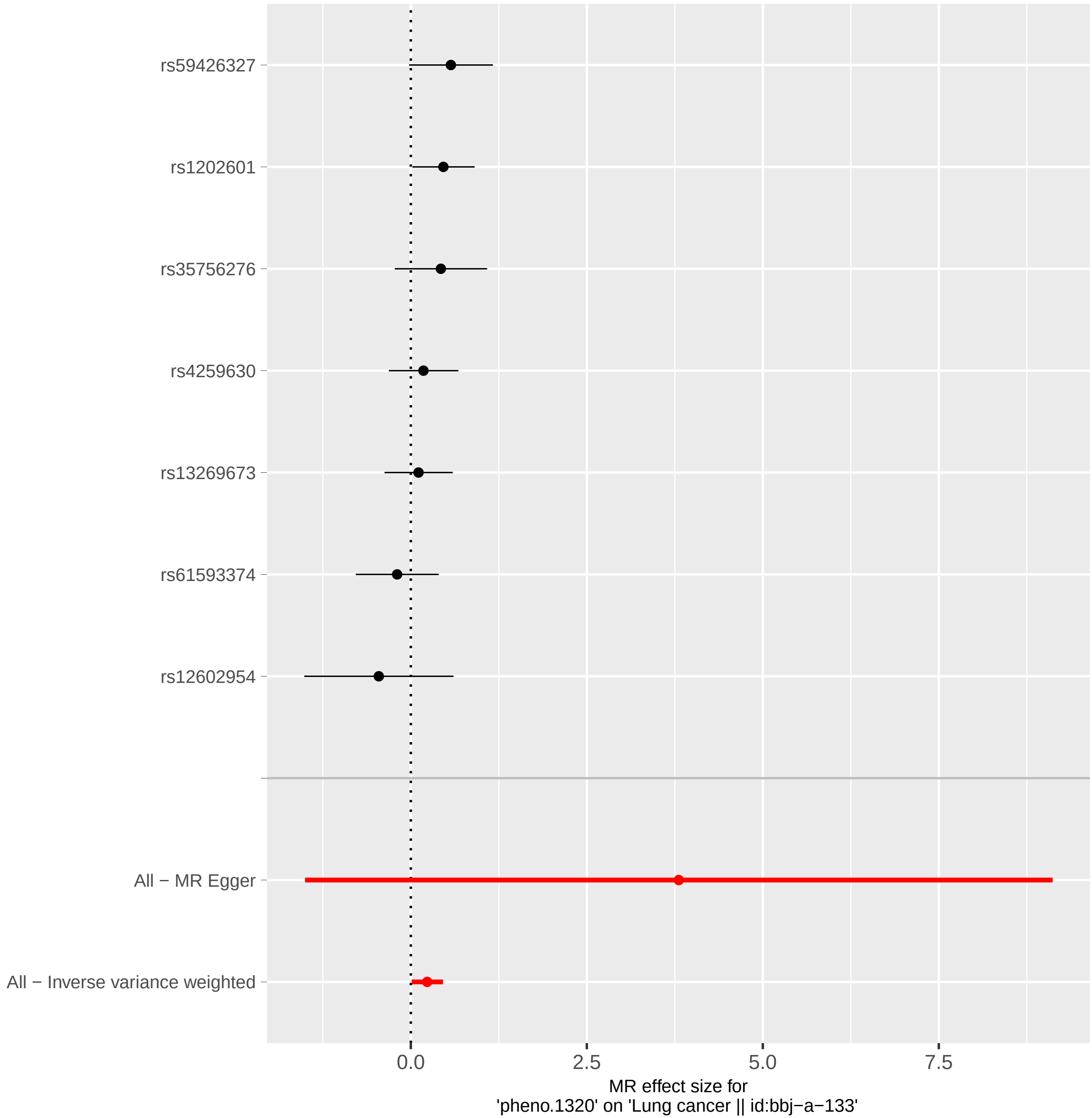

rs59426327
rs1202601
rs35756276
rs4259630
rs13269673
rs61593374
rs12602954
All − MR Egger
All − Inverse variance weighted
0.0
2.5
5.0
7.5
MR effect size for
'pheno.1320' on 'Lung cancer || id:bbj−a−133'
